# Dichlorido(1-{(*E*)-[phen­yl(pyridin-2-yl-κ*N*)methyl­idene]amino-κ*N*}pyrrolidin-2-one-κ*O*)copper(II) monohydrate

**DOI:** 10.1107/S1600536812035398

**Published:** 2012-08-23

**Authors:** Roji J. Kunnath, M.R. Prathapachandra Kurup, Seik Weng Ng

**Affiliations:** a Department of Applied Chemistry, Cochin University of Science and Technology, Kochi 682 022, India; b Department of Chemistry, University of Malaya, 50603 Kuala Lumpur, Malaysia; c Chemistry Department, King Abdulaziz University, PO Box 80203 Jeddah, Saudi Arabia

## Abstract

The Cu^II^ atom in the title compound, [CuCl_2_(C_16_H_15_N_3_O)]·H_2_O, is *N*,*N*′,*O*-chelated by the neutral Schiff base ligand and exists in a square-pyramidal geometry. It is displaced by 0.316 (1) Å out of the square plane (r.m.s. deviation = 0.015 Å) in the direction of the apical Cl atom. The apical Cl atoms of adjacent complex units are hydrogen-bond acceptors to two water mol­ecules, the inter­action generating a centrosymmetric dimer through a cyclic *R*_4_^2^(8) association.

## Related literature

For a history of Schiff bases, see: Tidwell (2008 ▶). For graph-set notation, see: Etter *et al.* (1990 ▶).
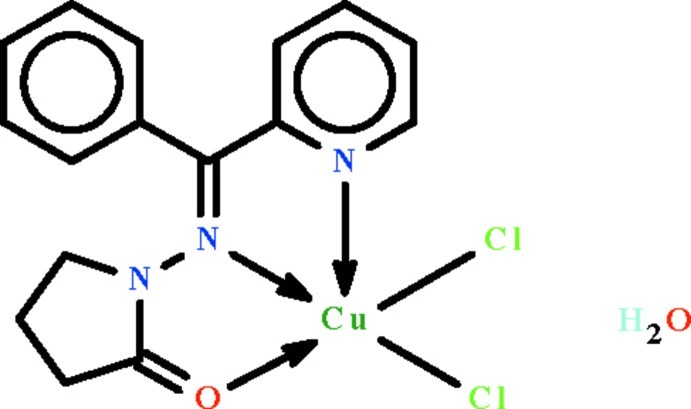


## Experimental

### 

#### Crystal data

[CuCl_2_(C_16_H_15_N_3_O)]·H_2_O
*M* *_r_* = 417.77
Triclinic, 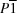

*a* = 9.1289 (2) Å
*b* = 9.4017 (2) Å
*c* = 10.6798 (2) Å
α = 90.4349 (6)°
β = 99.1627 (6)°
γ = 105.4911 (6)°
*V* = 870.84 (3) Å^3^
*Z* = 2
Mo *K*α radiation
μ = 1.57 mm^−1^
*T* = 293 K
0.30 × 0.30 × 0.30 mm

#### Data collection

Bruker Kappa APEXII CCD diffractometer
Absorption correction: multi-scan (*SADABS*; Sheldrick, 1996 ▶) *T*_min_ = 0.650, *T*_max_ = 0.650
14839 measured reflections
3987 independent reflections
3761 reflections with *I* > 2σ(*I*)
*R*_int_ = 0.033

#### Refinement

*R*[*F* ^2^ > 2σ(*F* ^2^)] = 0.026
*wR*(*F* ^2^) = 0.076
*S* = 1.07
3987 reflections
225 parameters
2 restraints
H atoms treated by a mixture of independent and constrained refinement
Δρ_max_ = 0.45 e Å^−3^
Δρ_min_ = −0.34 e Å^−3^

### 

Data collection: *APEX2* (Bruker, 2010 ▶); cell refinement: *SAINT* (Bruker, 2010 ▶); data reduction: *SAINT*; program(s) used to solve structure: *SHELXS97* (Sheldrick, 2008 ▶); program(s) used to refine structure: *SHELXL97* (Sheldrick, 2008 ▶); molecular graphics: *X-SEED* (Barbour, 2001 ▶); software used to prepare material for publication: *publCIF* (Westrip, 2010 ▶).

## Supplementary Material

Crystal structure: contains datablock(s) global, I. DOI: 10.1107/S1600536812035398/zs2227sup1.cif

Structure factors: contains datablock(s) I. DOI: 10.1107/S1600536812035398/zs2227Isup2.hkl

Additional supplementary materials:  crystallographic information; 3D view; checkCIF report

## Figures and Tables

**Table 1:** Hydrogen-bond geometry (Å, °)

*D*—H⋯*A*	*D*—H	H⋯*A*	*D*⋯*A*	*D*—H⋯*A*
O1w—H1⋯Cl1	0.83 (1)	2.34 (1)	3.175 (2)	178 (4)
O1w—H2⋯Cl1^i^	0.83 (1)	2.41 (2)	3.221 (3)	165 (4)

Symmetry code: (i) 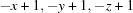.

**Fig. 1.**
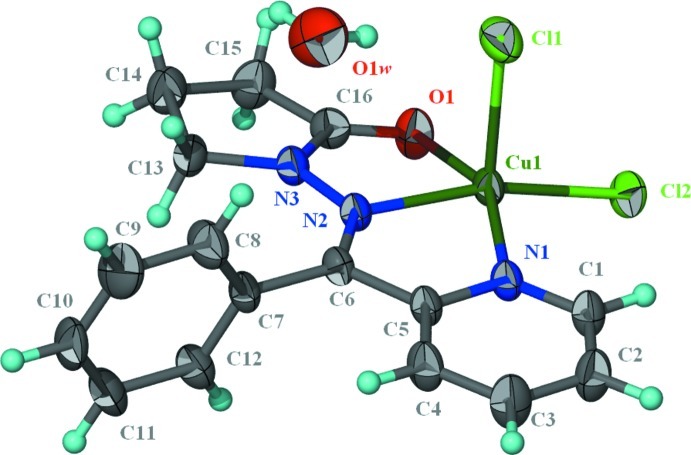
Thermal ellipsoid plot (Barbour, 2001) of [CuCl2(C16H15N3O)].H2O at the 50% probability level. Hydrogen atoms are drawn as spheres of arbitrary radius.

**Fig. 2.**
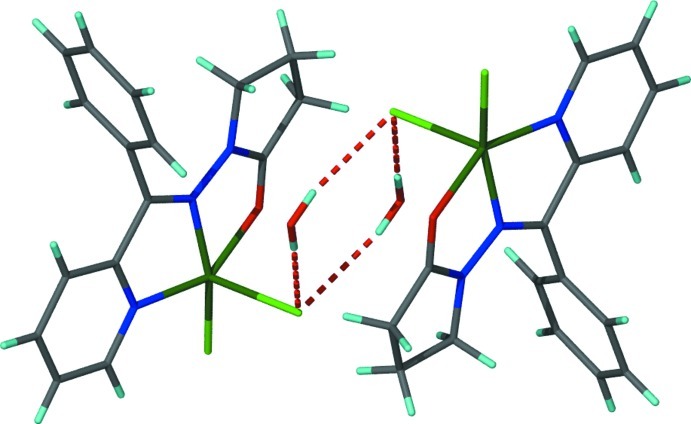
The hydrogen-bonded dimer.

## supplementary crystallographic information

### Comment

Among the plethora of Schiff bases that have been synthesized for the purpose of furnishing coordination compounds, some are synthesized *in situ*, and their formulation is inferred from the crystal structure of the product. Phenyl[(pyridin-2-yl)methylidene]amino]pyrrolidin-2-one is an example of such a Schiff base, which possess a carbonyl group and it forms a monohydrated complex with copper(II) chloride (Scheme I, Fig. 1). The Cu^II^ atom in this complex is *N*,*N*',*O*-chelated by the neutral Schiff ligand and has a square-pyramidal geometry, with the atom displaced out of the square plane in the direction of the apical Cl atom by 0.316 (1) Å. The apical Cl atoms of adjacent complex units are hydrogen-bond acceptors to two water molecules (Table 1), the interaction generating a centrosymmetric dimer (Fig. 2) through a cyclic *R*^2^_4_(8) association (Etter *et al.*, 1990).

### Experimental

1-[(*E*)-[Phenyl(pyridin-2-yl)methylidene]amino]pyrrolidin-2-one was synthesized *in situ* from 2-benzoylpyridine (0.183 g, 1 mmol) and 1-aminopyrrolidin-2-one (0.100 g, 1 mmol) by heating in methanol for 2 h. Copper(II) chloride dihydrate (0.170 g, 1 mmol) was added, and the mixture heated for 5 h. The resulting pale green solid was collected and recrystallized from methanol.

### Refinement

Carbon-bound H-atoms were placed in calculated positions (C—H = 0.93–0.97 Å) and were included in the refinement in the riding model approximation, with *U*_iso_(H) set to 1.2*U*_eq_(C). The water H-atoms were located in a difference Fourier map, and were refined with a distance restraint of O—H = 0.84±0.01 Å, with their displacement parameters refined. The (0 1 0) reflection was omitted owing to interference from the beam stop.

### Crystal data

**Table:**

[CuCl_2_(C_16_H_15_N_3_O)]·H_2_O	*Z* = 2
*M**_r_* = 417.77	*F*(000) = 426
Triclinic, *P*1	*D*_x_ = 1.593 Mg m^−^^3^
Hall symbol: -P 1	Mo *K*α radiation, λ = 0.71073 Å
*a* = 9.1289 (2) Å	Cell parameters from 9988 reflections
*b* = 9.4017 (2) Å	θ = 2.3–28.2°
*c* = 10.6798 (2) Å	µ = 1.57 mm^−^^1^
α = 90.4349 (6)°	*T* = 293 K
β = 99.1627 (6)°	Cube, green
γ = 105.4911 (6)°	0.30 × 0.30 × 0.30 mm
*V* = 870.84 (3) Å^3^	

### Data collection

**Table:**

Bruker Kappa APEXII CCD diffractometer	3987 independent reflections
Radiation source: fine-focus sealed tube	3761 reflections with *I* > 2σ(*I*)
Graphite monochromator	*R*_int_ = 0.033
ω scans	θ_max_ = 27.5°, θ_min_ = 2.8°
Absorption correction: multi-scan (*SADABS*; Sheldrick, 1996)	*h* = −11→11
*T*_min_ = 0.650, *T*_max_ = 0.650	*k* = −12→12
14839 measured reflections	*l* = −13→13

### Refinement

**Table:**

Refinement on *F*^2^	Primary atom site location: structure-invariant direct methods
Least-squares matrix: full	Secondary atom site location: difference Fourier map
*R*[*F*^2^ > 2σ(*F*^2^)] = 0.026	Hydrogen site location: inferred from neighbouring sites
*wR*(*F*^2^) = 0.076	H atoms treated by a mixture of independent and constrained refinement
*S* = 1.07	*w* = 1/[σ^2^(*F*_o_^2^) + (0.0379*P*)^2^ + 0.3259*P*] where *P* = (*F*_o_^2^ + 2*F*_c_^2^)/3
3987 reflections	(Δ/σ)_max_ = 0.001
225 parameters	Δρ_max_ = 0.45 e Å^−^^3^
2 restraints	Δρ_min_ = −0.34 e Å^−^^3^

### Fractional atomic coordinates and isotropic or equivalent isotropic displacement parameters (Å^2^)

**Table:**

	*x*	*y*	*z*	*U*_iso_*/*U*_eq_	
Cu1	0.48709 (2)	0.63969 (2)	0.845334 (18)	0.03229 (8)	
Cl1	0.54227 (8)	0.72830 (6)	0.64075 (5)	0.06303 (16)	
Cl2	0.64240 (5)	0.82061 (5)	0.97595 (4)	0.04703 (12)	
O1	0.62811 (14)	0.49646 (15)	0.86442 (14)	0.0478 (3)	
O1W	0.2859 (2)	0.5064 (3)	0.4420 (2)	0.0832 (6)	
N1	0.28316 (15)	0.68570 (15)	0.84571 (13)	0.0340 (3)	
N2	0.33922 (15)	0.44928 (14)	0.77515 (13)	0.0308 (3)	
N3	0.40965 (15)	0.34475 (15)	0.75166 (14)	0.0339 (3)	
C1	0.2610 (2)	0.81241 (19)	0.88367 (18)	0.0410 (4)	
H1A	0.3462	0.8893	0.9176	0.049*	
C2	0.1151 (2)	0.8336 (2)	0.8743 (2)	0.0505 (5)	
H2A	0.1028	0.9227	0.9030	0.061*	
C3	−0.0106 (2)	0.7216 (2)	0.8222 (2)	0.0557 (5)	
H3	−0.1094	0.7339	0.8150	0.067*	
C4	0.0108 (2)	0.5900 (2)	0.7803 (2)	0.0467 (4)	
H4	−0.0729	0.5129	0.7439	0.056*	
C5	0.15940 (18)	0.57554 (17)	0.79400 (16)	0.0333 (3)	
C6	0.19396 (18)	0.43781 (17)	0.75440 (15)	0.0320 (3)	
C7	0.06742 (18)	0.30651 (17)	0.70149 (17)	0.0342 (3)	
C8	0.0047 (3)	0.2941 (2)	0.5739 (2)	0.0549 (5)	
H8	0.0428	0.3672	0.5202	0.066*	
C9	−0.1152 (3)	0.1720 (3)	0.5270 (2)	0.0704 (7)	
H9	−0.1575	0.1632	0.4412	0.085*	
C10	−0.1720 (2)	0.0643 (2)	0.6054 (3)	0.0614 (6)	
H10	−0.2518	−0.0179	0.5726	0.074*	
C11	−0.1118 (2)	0.0772 (2)	0.7324 (2)	0.0530 (5)	
H11	−0.1516	0.0041	0.7856	0.064*	
C12	0.0085 (2)	0.1990 (2)	0.7818 (2)	0.0428 (4)	
H12	0.0491	0.2082	0.8680	0.051*	
C13	0.3480 (2)	0.19716 (19)	0.68807 (18)	0.0399 (4)	
H13A	0.2971	0.2019	0.6019	0.048*	
H13B	0.2759	0.1326	0.7344	0.048*	
C14	0.4930 (2)	0.1453 (2)	0.6898 (2)	0.0510 (5)	
H14A	0.4770	0.0461	0.7198	0.061*	
H14B	0.5182	0.1444	0.6049	0.061*	
C15	0.6226 (2)	0.2524 (2)	0.7783 (2)	0.0509 (5)	
H15A	0.6500	0.2064	0.8563	0.061*	
H15B	0.7132	0.2849	0.7382	0.061*	
C16	0.5608 (2)	0.3787 (2)	0.80431 (18)	0.0392 (4)	
H1	0.355 (3)	0.564 (3)	0.493 (3)	0.100 (12)*	
H2	0.330 (4)	0.441 (3)	0.435 (4)	0.114 (15)*	

### Atomic displacement parameters (Å^2^)

**Table:**

	*U*^11^	*U*^22^	*U*^33^	*U*^12^	*U*^13^	*U*^23^
Cu1	0.02431 (11)	0.02906 (11)	0.03759 (12)	0.00025 (8)	0.00019 (7)	−0.00478 (8)
Cl1	0.0821 (4)	0.0500 (3)	0.0433 (3)	−0.0090 (3)	0.0168 (2)	0.0018 (2)
Cl2	0.0366 (2)	0.0455 (2)	0.0467 (2)	−0.00346 (18)	−0.00297 (17)	−0.01499 (18)
O1	0.0300 (6)	0.0431 (7)	0.0650 (9)	0.0087 (5)	−0.0048 (6)	−0.0102 (6)
O1W	0.0539 (11)	0.1031 (17)	0.0784 (13)	0.0071 (11)	−0.0049 (9)	−0.0176 (12)
N1	0.0284 (6)	0.0284 (6)	0.0404 (7)	0.0027 (5)	0.0005 (5)	−0.0031 (5)
N2	0.0270 (6)	0.0253 (6)	0.0374 (7)	0.0043 (5)	0.0027 (5)	−0.0006 (5)
N3	0.0292 (6)	0.0277 (6)	0.0426 (7)	0.0055 (5)	0.0039 (5)	−0.0018 (5)
C1	0.0379 (9)	0.0302 (8)	0.0495 (10)	0.0047 (7)	−0.0003 (7)	−0.0060 (7)
C2	0.0452 (10)	0.0355 (9)	0.0704 (13)	0.0149 (8)	0.0020 (9)	−0.0109 (9)
C3	0.0357 (9)	0.0442 (10)	0.0866 (16)	0.0148 (8)	0.0026 (10)	−0.0097 (10)
C4	0.0281 (8)	0.0363 (9)	0.0696 (12)	0.0042 (7)	−0.0010 (8)	−0.0078 (8)
C5	0.0274 (7)	0.0270 (7)	0.0409 (8)	0.0024 (6)	0.0011 (6)	−0.0019 (6)
C6	0.0281 (7)	0.0272 (7)	0.0357 (8)	0.0016 (6)	0.0013 (6)	−0.0006 (6)
C7	0.0244 (7)	0.0259 (7)	0.0487 (9)	0.0030 (6)	0.0022 (6)	−0.0033 (6)
C8	0.0518 (12)	0.0488 (11)	0.0482 (11)	−0.0085 (9)	0.0008 (9)	−0.0049 (9)
C9	0.0602 (14)	0.0709 (16)	0.0591 (14)	−0.0100 (12)	−0.0031 (11)	−0.0230 (12)
C10	0.0396 (10)	0.0414 (11)	0.0903 (17)	−0.0088 (8)	0.0091 (10)	−0.0261 (11)
C11	0.0368 (9)	0.0295 (8)	0.0926 (16)	0.0040 (7)	0.0193 (10)	0.0060 (9)
C12	0.0334 (8)	0.0344 (8)	0.0578 (11)	0.0056 (7)	0.0058 (7)	0.0058 (7)
C13	0.0404 (9)	0.0314 (8)	0.0460 (9)	0.0073 (7)	0.0060 (7)	−0.0074 (7)
C14	0.0479 (11)	0.0396 (10)	0.0684 (13)	0.0156 (8)	0.0119 (9)	−0.0043 (9)
C15	0.0423 (10)	0.0509 (11)	0.0632 (12)	0.0225 (9)	0.0033 (9)	−0.0043 (9)
C16	0.0321 (8)	0.0392 (9)	0.0461 (9)	0.0102 (7)	0.0048 (7)	0.0014 (7)

### Geometric parameters (Å, º)

**Table:**

Cu1—N2	1.9888 (13)	C5—C6	1.486 (2)
Cu1—N1	2.0213 (14)	C6—C7	1.482 (2)
Cu1—O1	2.0878 (13)	C7—C8	1.382 (3)
Cu1—Cl2	2.2125 (4)	C7—C12	1.384 (2)
Cu1—Cl1	2.4240 (5)	C8—C9	1.383 (3)
O1—C16	1.231 (2)	C8—H8	0.9300
O1W—H1	0.834 (10)	C9—C10	1.366 (4)
O1W—H2	0.833 (10)	C9—H9	0.9300
N1—C1	1.331 (2)	C10—C11	1.372 (4)
N1—C5	1.350 (2)	C10—H10	0.9300
N2—C6	1.284 (2)	C11—C12	1.389 (3)
N2—N3	1.3518 (19)	C11—H11	0.9300
N3—C16	1.355 (2)	C12—H12	0.9300
N3—C13	1.466 (2)	C13—C14	1.526 (3)
C1—C2	1.387 (3)	C13—H13A	0.9700
C1—H1A	0.9300	C13—H13B	0.9700
C2—C3	1.371 (3)	C14—C15	1.517 (3)
C2—H2A	0.9300	C14—H14A	0.9700
C3—C4	1.385 (3)	C14—H14B	0.9700
C3—H3	0.9300	C15—C16	1.486 (2)
C4—C5	1.384 (2)	C15—H15A	0.9700
C4—H4	0.9300	C15—H15B	0.9700
			
N2—Cu1—N1	78.77 (5)	C8—C7—C12	120.20 (17)
N2—Cu1—O1	78.29 (5)	C8—C7—C6	120.33 (16)
N1—Cu1—O1	152.34 (6)	C12—C7—C6	119.42 (16)
N2—Cu1—Cl2	163.34 (4)	C7—C8—C9	119.4 (2)
N1—Cu1—Cl2	100.41 (4)	C7—C8—H8	120.3
O1—Cu1—Cl2	97.17 (4)	C9—C8—H8	120.3
N2—Cu1—Cl1	95.08 (4)	C10—C9—C8	120.6 (2)
N1—Cu1—Cl1	100.24 (4)	C10—C9—H9	119.7
O1—Cu1—Cl1	96.99 (5)	C8—C9—H9	119.7
Cl2—Cu1—Cl1	101.40 (2)	C9—C10—C11	120.19 (19)
C16—O1—Cu1	109.91 (11)	C9—C10—H10	119.9
H1—O1W—H2	98 (3)	C11—C10—H10	119.9
C1—N1—C5	118.68 (14)	C10—C11—C12	120.2 (2)
C1—N1—Cu1	127.24 (12)	C10—C11—H11	119.9
C5—N1—Cu1	113.95 (11)	C12—C11—H11	119.9
C6—N2—N3	127.33 (14)	C7—C12—C11	119.36 (19)
C6—N2—Cu1	119.74 (11)	C7—C12—H12	120.3
N3—N2—Cu1	112.90 (10)	C11—C12—H12	120.3
N2—N3—C16	113.89 (13)	N3—C13—C14	102.37 (15)
N2—N3—C13	131.06 (14)	N3—C13—H13A	111.3
C16—N3—C13	114.85 (14)	C14—C13—H13A	111.3
N1—C1—C2	122.22 (16)	N3—C13—H13B	111.3
N1—C1—H1A	118.9	C14—C13—H13B	111.3
C2—C1—H1A	118.9	H13A—C13—H13B	109.2
C3—C2—C1	119.11 (17)	C15—C14—C13	107.37 (15)
C3—C2—H2A	120.4	C15—C14—H14A	110.2
C1—C2—H2A	120.4	C13—C14—H14A	110.2
C2—C3—C4	119.32 (18)	C15—C14—H14B	110.2
C2—C3—H3	120.3	C13—C14—H14B	110.2
C4—C3—H3	120.3	H14A—C14—H14B	108.5
C5—C4—C3	118.56 (17)	C16—C15—C14	105.17 (15)
C5—C4—H4	120.7	C16—C15—H15A	110.7
C3—C4—H4	120.7	C14—C15—H15A	110.7
N1—C5—C4	122.09 (15)	C16—C15—H15B	110.7
N1—C5—C6	115.32 (14)	C14—C15—H15B	110.7
C4—C5—C6	122.59 (15)	H15A—C15—H15B	108.8
N2—C6—C7	127.44 (14)	O1—C16—N3	122.62 (16)
N2—C6—C5	112.02 (13)	O1—C16—C15	128.59 (17)
C7—C6—C5	120.52 (13)	N3—C16—C15	108.77 (15)
			
N2—Cu1—O1—C16	12.11 (13)	C3—C4—C5—C6	178.79 (19)
N1—Cu1—O1—C16	46.6 (2)	N3—N2—C6—C7	1.0 (3)
Cl2—Cu1—O1—C16	175.86 (13)	Cu1—N2—C6—C7	179.23 (13)
Cl1—Cu1—O1—C16	−81.66 (13)	N3—N2—C6—C5	179.28 (14)
N2—Cu1—N1—C1	−179.79 (16)	Cu1—N2—C6—C5	−2.54 (19)
O1—Cu1—N1—C1	145.74 (15)	N1—C5—C6—N2	−1.0 (2)
Cl2—Cu1—N1—C1	17.15 (16)	C4—C5—C6—N2	179.64 (17)
Cl1—Cu1—N1—C1	−86.58 (15)	N1—C5—C6—C7	177.33 (15)
N2—Cu1—N1—C5	−3.96 (12)	C4—C5—C6—C7	−2.0 (3)
O1—Cu1—N1—C5	−38.43 (19)	N2—C6—C7—C8	−97.6 (2)
Cl2—Cu1—N1—C5	−167.02 (11)	C5—C6—C7—C8	84.3 (2)
Cl1—Cu1—N1—C5	89.24 (12)	N2—C6—C7—C12	84.8 (2)
N1—Cu1—N2—C6	3.67 (13)	C5—C6—C7—C12	−93.27 (19)
O1—Cu1—N2—C6	168.11 (14)	C12—C7—C8—C9	−1.3 (3)
Cl2—Cu1—N2—C6	92.48 (18)	C6—C7—C8—C9	−178.8 (2)
Cl1—Cu1—N2—C6	−95.78 (13)	C7—C8—C9—C10	0.2 (4)
N1—Cu1—N2—N3	−177.89 (12)	C8—C9—C10—C11	0.8 (4)
O1—Cu1—N2—N3	−13.46 (11)	C9—C10—C11—C12	−0.7 (3)
Cl2—Cu1—N2—N3	−89.09 (17)	C8—C7—C12—C11	1.4 (3)
Cl1—Cu1—N2—N3	82.65 (11)	C6—C7—C12—C11	178.96 (16)
C6—N2—N3—C16	−168.57 (16)	C10—C11—C12—C7	−0.4 (3)
Cu1—N2—N3—C16	13.14 (17)	N2—N3—C13—C14	177.20 (17)
C6—N2—N3—C13	5.9 (3)	C16—N3—C13—C14	−8.4 (2)
Cu1—N2—N3—C13	−172.38 (14)	N3—C13—C14—C15	11.8 (2)
C5—N1—C1—C2	1.4 (3)	C13—C14—C15—C16	−11.4 (2)
Cu1—N1—C1—C2	177.05 (15)	Cu1—O1—C16—N3	−9.2 (2)
N1—C1—C2—C3	−1.2 (3)	Cu1—O1—C16—C15	172.06 (17)
C1—C2—C3—C4	0.1 (4)	N2—N3—C16—O1	−2.2 (3)
C2—C3—C4—C5	0.7 (3)	C13—N3—C16—O1	−177.64 (17)
C1—N1—C5—C4	−0.5 (3)	N2—N3—C16—C15	176.76 (15)
Cu1—N1—C5—C4	−176.76 (15)	C13—N3—C16—C15	1.4 (2)
C1—N1—C5—C6	−179.87 (15)	C14—C15—C16—O1	−174.7 (2)
Cu1—N1—C5—C6	3.92 (18)	C14—C15—C16—N3	6.4 (2)
C3—C4—C5—N1	−0.5 (3)		

### Hydrogen-bond geometry (Å, º)

**Table:**

*D*—H···*A*	*D*—H	H···*A*	*D*···*A*	*D*—H···*A*
O1w—H1···Cl1	0.83 (1)	2.34 (1)	3.175 (2)	178 (4)
O1w—H2···Cl1^i^	0.83 (1)	2.41 (2)	3.221 (3)	165 (4)

Symmetry code: (i) −*x*+1, −*y*+1, −*z*+1.
